# The Influence of Entrepreneurial Characteristics on the Performance of Tourism Vlogger Entrepreneurs

**DOI:** 10.3389/fpsyg.2021.725545

**Published:** 2021-08-09

**Authors:** Qing Xie, Lisha Liu, Haider Malik, Supat Chupradit, Priyanut Wutti Chupradit

**Affiliations:** ^1^School of Business and Economics, Universiti Putra Malaysia, Seri Kembangan, Malaysia; ^2^School of Economics and Management, Anshun University, Anshun, China; ^3^Ecological Environment Monitoring Center of Chengdu, Chengdu, China; ^4^National University of Computer and Emerging Sciences, Islamabad, Pakistan; ^5^Department of Occupational Therapy, Faculty of Associated Medical Sciences, Chiang Mai University, Chiang Mai, Thailand; ^6^Educational Psychology and Guidance, Department of Educational Foundations and Development, Faculty of Education, Chiang Mai University, Chiang Mai, Thailand

**Keywords:** vloggers, entrepreneurial characteristics, entrepreneurial intentions, organizational performance, performance

## Abstract

The current Internet revolution has changed the entrepreneurial opportunities and trends. This study explores the relationship between entrepreneurial characteristics (e.g., innovation, leadership, planning, and sociability) and the performance of entrepreneurial vloggers in India. In addition, this study considers the mediating effect of entrepreneurial intentions. This study is cross-sectional, and it considered 128 entrepreneurial vloggers for the analysis. The SmartPLS application was used to estimate the structural equation modeling (SEM) analysis for the estimation of reliability and validity along with the path relationship. The findings are more important as the entrepreneurial characteristics can meaningfully predict the performance of entrepreneurial vloggers in a positive direction. Moreover, the relationship between entrepreneurial characteristics and the performance of entrepreneurs is partially mediated by entrepreneurial intentions. These findings have important implications for vloggers in Indian or other countries with similar nature. This study has also put some future research directions at the end.

## Introduction

Entrepreneurship has always been embraced by many decision-makers and national governments as a key development factor because it increases the social welfare through its favorable impact on innovation, job creation, and economic growth (Al Mamun et al., 2018; Cai et al., 2018). In societies, current dynamic trends, such as rapid changes in the environment and rising cognitive complexities in our lives, have led to a shifting nature of interaction among individuals and groups. Therefore, in this situation, the entrepreneurial characteristics play a significant role, not only for those people who want to start up a career as an owner–manager in various environments but also for those who want to follow a career as an entrepreneur (Holienka et al., 2015). Moreover, the entrepreneurial characteristics could be nurtured through entrepreneurial education because it can directly influence the intentions of an enterpriser to start up a business venture (Thoyib et al., 2016).

In the tourism industry, the entrepreneurial characteristics have been an area of emerging interest in the last two decades (Menon and Uddin, 2010; Solvoll et al., 2015). Tourism has traditionally been perceived as a fertile ground for entrepreneurs due to the prevalence of small businesses and low entry barriers (Li and Giles, 2015; Nikraftar and Hosseini, 2016). In recent years, evolution has been predicted in travel agencies due to the benefits of social media, explorers, guides, vloggers, and innovation in technology (Birch-Jensen, 2020). Moreover, the rapid development and demand of digital technology have encouraged the emergence of social media sites due to their open-access services and web-based community. Social media has become a popular platform for vloggers; they can share their content in the form of photos and video clips. Thus, they can easily attract and organize trips for the tourists by providing them with convenient information (Chen and Dermawan, 2020).

An entrepreneur who has entrepreneurial characteristics leads to a preference for entrepreneurial activities. The entrepreneurial intention is a cognitive model of actions implemented by individuals to start a new venture or add value to an existing business (Fini et al., 2012). This study focused on the entrepreneurial intentions implemented by individuals to launch a new business. In the literature, most of the models of entrepreneurial intentions have been analyzed by the theory of planned behavior (TPB) proposed by Ajzen (1991). The main purpose of this study is to highlight the performance of tourism vloggers through the mediating role of entrepreneurship intentions. Furthermore, it is imperative to investigate which entrepreneurship characteristics affect the entrepreneurial intentions of tourists for the establishment of new businesses.

In the current scenario, we focused on the performance of tourism vloggers in India. As He et al. (2020) argued, in the last three decades, India has witnessed remarkable entrepreneurship development. A strand of literature has explored different entrepreneurial characteristics such as locus of control (Bonnett and Furnham, 1991; Rashid et al., 2003; Hargreaves et al., 2008; Othman and Ishak, 2009; Al Mamun et al., 2018), risk-taking (Sexton and Bowman, 1985; Yusof et al., 2007), social networking (Taormina and Lao, 2007; Yang et al., 2013), self-efficacy (Robinson and Sexton, 1994; Chen et al., 2006; Pittaway et al., 2011; Zellweger et al., 2011; Piperopoulos and Dimov, 2015), creativity (Hargreaves et al., 2008) in the context of student entrepreneurship, while only limited studies have examined these entrepreneurial characteristics such as sociability (Markman and Baron, 2003; Schmidt and Bohnenberger, 2009; Rocha, 2014), leadership (Dawson et al., 2000; Schmidt and Bohnenberger, 2009), planning (Schmidt and Bohnenberger, 2009; Rocha, 2014), innovation (Reneau et al., 1989; Schmidt and Bohnenberger, 2009; Rocha, 2014), and their impact on the intentions of enterpriser and business performance. Therefore, this study fills this gap by considering these entrepreneurial characteristics as the mediating role of entrepreneurial intentions and their impact on the performance of tourism vloggers.

The remaining structure of the study is as follows: the “Literature review” section provides prior research literature on considered constructs. The section “Research methodology” employed to test the hypothesis. The sections “Data analysis and results” and “Discussion of findings” are related to the interpretation of our empirical study. The last section, “Conclusion” concludes the study by offering implications and future recommendations.

## Literature Review

### Entrepreneurship Characteristics

The entrepreneurial characteristics are those personality traits that mark a person as an entrepreneur (Kazanjian et al., 2001; Maritan and Brush, 2003), while they are specifically defined as the abilities of an enterpriser to identify new business opportunities and to take initiatives by utilizing them in competitive environmental situations (Velichová, 2013). In the earlier literature, many researchers have devoted much attention to figure out which characteristics are essential for an entrepreneur personality to launch a new business; even now, these characters are under-debated among economists and entrepreneurship scholars in the literature as no universal consensus has been defined (Rashid et al., 2003; Zimmerer et al., 2008; Sunarya, 2013; Thoyib et al., 2016).

According to the study by Falola et al. (2018), the entrepreneurial characteristics are vitally important for the establishment of a business because individuals combine their characteristics with the innovative capabilities of the company for achieving success. Gürol and Atsan (2006) argued that entrepreneurial characteristics are a combination of individual, social, and environmental factors. Individual aspects include personal characteristics and values; social factors consist of family, societal, and professional backgrounds, while environmental factors are extrinsic characteristics (Hsieh et al., 2019). All these characteristics influence the objectives and strategic actions of a firm, which in turn affect the performance of a business (Frese et al., 2012). Thus, in this study, we examined the following entrepreneurial characteristics: sociability, planning, leadership, and innovation and their impact on the intentions of the tourism vlogger and their performance.

#### Sociability

The sociability definitions have been varied across various technology-mediated situations (Phua et al., 2017). Wu et al. (2010) argued that sociability is the ability of an individual to engage with others and examined how it influences the private experiences and behavior of the user, whereas in the study by Wang et al. (2015) sociability is termed as the tendency of a person to engage with others, which is seen to be a crucial driver for the creation and preservation of cooperation. Rocha (2014) defined sociability as the extent to which people utilize social media to assist their professional activities. Earlier studies proposed the determinants of sociability as several participants, new users joining every month, errors, messages delivered as well as the satisfaction of the member, productivity, retention, trustworthiness, and other similar quantitative indicators (Kim et al., 2016). According to Chen and Dermawan (2020), sociability is a cognitive capital, and its main components are people, purpose, policies, social climate, self-representation, and assistance with formal interaction. This shows that sociability has a direct influence on the entrepreneurial intentions of vloggers to develop a new business, and it can easily attract tourists by sharing informative content on social media sites.

#### Planning

Planning is related to what to do and how to do it as it assists the entrepreneur in initiating, maintaining, and evaluating the actions required for achieving desired goals and objectives of a new business (Frese et al., 2012). It is often accomplished by the systemization of ideas and written documents, which anticipates the future of enterprisers (Testa and Frascheri, 2015). According to Delmar and Shane (2003) and Santos et al. (2012), business planning is a guiding tool that assists the entrepreneur in taking management decisions. Moreover, Brinckmann et al. (2010) argued that proper planning has a positive impact on the performance of businesses. In the literature, limited studies have documented the business planning of a tourism vlogger and its impact on their performance, but according to general understating, planning is essential for the establishment and development of a new business (Battistelli et al., 2014). Through good planning, vloggers can satisfy their customers by organizing a pleasant trip according to their preferences; this will ultimately increase the performance of their business.

#### Leadership

Leadership is one of the most important characters for an entrepreneur, and the objective of leaders is to improve their professional environment while utilizing the existing entrepreneurial resources to achieve efficiency and innovation (Piperopoulos and Dimov, 2015). Dunne et al. (2016) argued that an effective leader in entrepreneurship is a key determinant of organizational success or failure. Prior empirical studies have concluded that leadership is an essential entrepreneur character and recursively linked with each other (Harrison et al., 2016; Leitch and Volery, 2017). Harrison et al. (2016) discussed the importance of understanding entrepreneurs who are characterized by leadership since they are continually confronted with uncertainty and risk. Similarly, Leitch and Volery (2017) indicated that entrepreneurs must be prepared with leadership skills to identify and utilize opportunities. Therefore, the earlier discussion confirmed that the leadership character in tourism entrepreneurs plays a dominant role in enhancing the performance of the vloggers.

#### Innovation

Innovation is a complicated process that frequently demands knowledge and expertise from other economies (Bergenholtz and Waldstrøm, 2011). Due to the emergence of unique and powerful digital platforms, technologies, and infrastructures, the ways of innovation and entrepreneurship have significantly been transformed (Abbas et al., 2019). Prior few studies (Landström et al., 2015; Ribeiro et al., 2020) argued that innovation and entrepreneurship have a strong relationship with each other, while others documented that despite these evident links, innovation, and entrepreneurship are two distinct phenomena because not all the enterprisers are innovative and not all available information leads to a successful entrepreneurship (Landström et al., 2015; Malerba and McKelvey, 2020). Therefore, it is necessary that entrepreneurs should give greater attention to the innovation process for attaining competitive advantages. Similarly, innovation processes like advanced technologies have a significant impact on the intentions and performance of tourism vloggers. Thus, vloggers can easily offer information on the tourist destination by uploading relevant content on social media sites.

### Entrepreneurial Intentions

Identifying the factors that influence the entrepreneurial intentions regarding launching a new business has always been a vital concern for researchers and policymakers (Miller et al., 2009). Entrepreneurial intentions are defined as a conscious state of mind that proceeds actions and directs attention toward achieving specific goals (Bird and Jelinek, 1989; Santos et al., 2012; Roy et al., 2017). Thus, intentions can be considered as a measure of willingness of one to do something or a measure of how much effort is used to achieve a specific objective. Studies documented that motivation and determination are needed to transform entrepreneurial ideas into reality (Delmar and Shane, 2003). One who has better entrepreneurial intentions can easily perform entrepreneurial activities and can also be motivated to launch and develop a new business (Drnovsek and Erikson, 2005), therefore, seeming important to know entrepreneur intentions as these have a significant impact on the overall performance of a business.

Earlier studies have focused on several personal and environmental factors that influence the intentions of the entrepreneur (Bird and Jelinek, 1989; Krueger et al., 2000; Delmar and Shane, 2003; Indarti and Rostiani, 2008). Entrepreneurial intentions are also influenced by the entrepreneur programs, which provide information and best skills to an entrepreneur through training programs (Hytti and O'Gorman, 2004; Olugbola, 2017; Mahto and McDowell, 2018). Moreover, Kolvereid and Isaksen (2006) found that entrepreneurs who participated in entrepreneurship programs and activities displayed significant entrepreneurial attitude and intentions than others who did not attend such training programs. Furthermore, it was noted that these individuals are more creative, capable of encouraging others, analytical, more competent to take initiatives, better networking, and can handle difficult situations easily.

The models of entrepreneur intention assist an individual in understanding and predicting his/her behavior, as well as explain how entrepreneurs perceive opportunities by analyzing their intentions and other influencing variables that affect their intentions (Krueger et al., 2000; Shepherd and Krueger, 2002). According to TPB, theory intentions are defined as a key determinant of individual behavior, so understanding the individual behavior through intentions provides a better insight to the entrepreneur for the process of venture creation (Galanakis and Giourka, 2017). Thus, intention-based models are extremely useful for entrepreneurship research because entrepreneurial activities are planned behavior, and understanding intentions helps the entrepreneur to detect prospective actions. Similar to these studies (Miralles et al., 2016), this study mainly focuses on the intentions because entrepreneurial vloggers are still acquiring knowledge that will help them to launch a new business and performing their business career effectively.

### Entrepreneurship Performance

Entrepreneurship is one of the key factors that lead to a successful business performance even under extreme unpredictable situations (Cho and Lee, 2018). Business performance is termed as how an organization can achieve its desired set of goals by coping with other fluctuating factors (Cho and Lee, 2018). Moreover, entrepreneurs can attain their best performance by fulfilling the need and satisfying their customers. A strand of literature has documented the association between entrepreneurial characteristics and entrepreneurship performance (Robinson and Sexton, 1994; Kiggundu, 2002; Falola et al., 2018) and found that a strong relationship exists between entrepreneurial characteristics and performance of a venture. More entrepreneur tourism vloggers possess entrepreneurial performance which is significantly influenced by the entrepreneurial characteristics such as innovation, sociability, leadership, and planning (Markman and Baron, 2003; Schmidt and Bohnenberger, 2009; Rocha, 2014). Many studies have considered the performance of a vlogger in other areas such as fashion designing and purchasing products, while only few studies have documented in the domain of travel vloggers (Choi and Lee, 2019) provided evidence of how vloggers talk to an audience similar to celebrities as vloggers can build a relationship with their viewers through online friendship. In this way, innovative technologies and social media sites provide a platform for vloggers; they can easily upload their content and can attract viewers toward them through any opinion leader (Lee and Watkins, 2016).

According to Safko (2010), vlogging is an efficient form of communication because viewers can observe emotions, body language, and tone through video clips. Shared content on websites can also influence the attitude of the audience, if viewers show positive intentions regarding the uploaded content, then content will be more exposed to social media (Mangold and Faulds, 2009). Recently, Malerba and McKelvey (2020) argued that vloggers have a more influential impact on the products of the customer experience as compared with search products.

### Entrepreneurial Characteristics and Intentions

Entrepreneurial characteristics play an important role in determining individual behavior (Tran and Von Korflesch, 2016). Earlier researchers concluded that there exists a positive association between the characteristics of entrepreneurs and their intentions (Zhao and Seibert, 2006). According to Mahto and McDowell (2018), the entrepreneurial characteristics are vitally important for the establishment of business because individuals combine their characteristics with the innovative capabilities of company for achieving success. This study has explored the four main characteristics of an entrepreneur, namely, sociability, planning, leadership, and innovation, which have a significant impact on the intentions of an entrepreneur (Wang et al., 2015). Dhaundiyal and Coughlan (2016) found that a higher sociability character has a more influential impact on the intentions of an entrepreneur as a sociable person looks more forward toward opportunities and encourages social interaction. While in the case of leadership, empirical studies have concluded that leadership is an essential entrepreneur character, and both are recursively linked with each other (Harrison et al., 2016; Leitch and Volery, 2017). In the literature, limited studies have documented business planning of tourism vloggers and its impact on their entrepreneurial intentions. Leitch and Volery (2017) indicated that entrepreneurs must be prepared with leadership skills to identify and utilize opportunities. However, the innovative characteristics have a dominant impact on the intentions of an entrepreneur. Prior few studies (Landström et al., 2015; Ribeiro et al., 2020) argued that innovation and entrepreneurship have a strong relationship with each other, while others and vloggers can satisfy tourists by the usage of innovative technologies. The earlier discussion shows that there exists a significant association between environmental characteristics and their intentions.

### Entrepreneurial Intentions and Performance

Entrepreneurial intentions have an influential impact on the overall performance of a business as an entrepreneur who has more entrepreneurial intentions can easily perform the entrepreneurial activities and also gets motivated to launch and develop a new business (Drnovsek and Erikson, 2005). Earlier studies (Bird and Jelinek, 1989; Krueger et al., 2000; Delmar and Shane, 2003; Indarti and Rostiani, 2008) documented that entrepreneurial intentions have a positive association with business performance. Entrepreneurial intentions are also influenced by the entrepreneur programs as these training programs provide information and best skills to the entrepreneur, which ultimately leads to business success (Olugbola, 2017; Mahto and McDowell, 2018). Entrepreneurial vloggers can satisfy their customers by sharing contents that will positively facilitate them. A strand of literature (Robinson and Sexton, 1994; Kiggundu, 2002) has found that a strong relationship exists between entrepreneur intentions and the performance of a venture. This study has documented that entrepreneur vloggers can attract tourists through sharing photos and clips on their social media profiles, which will influence their ultimate performance. Overall, prior studies indicate that there exists a strong relationship between entrepreneurial intentions of vloggers and their performance.

**Figure 1:**
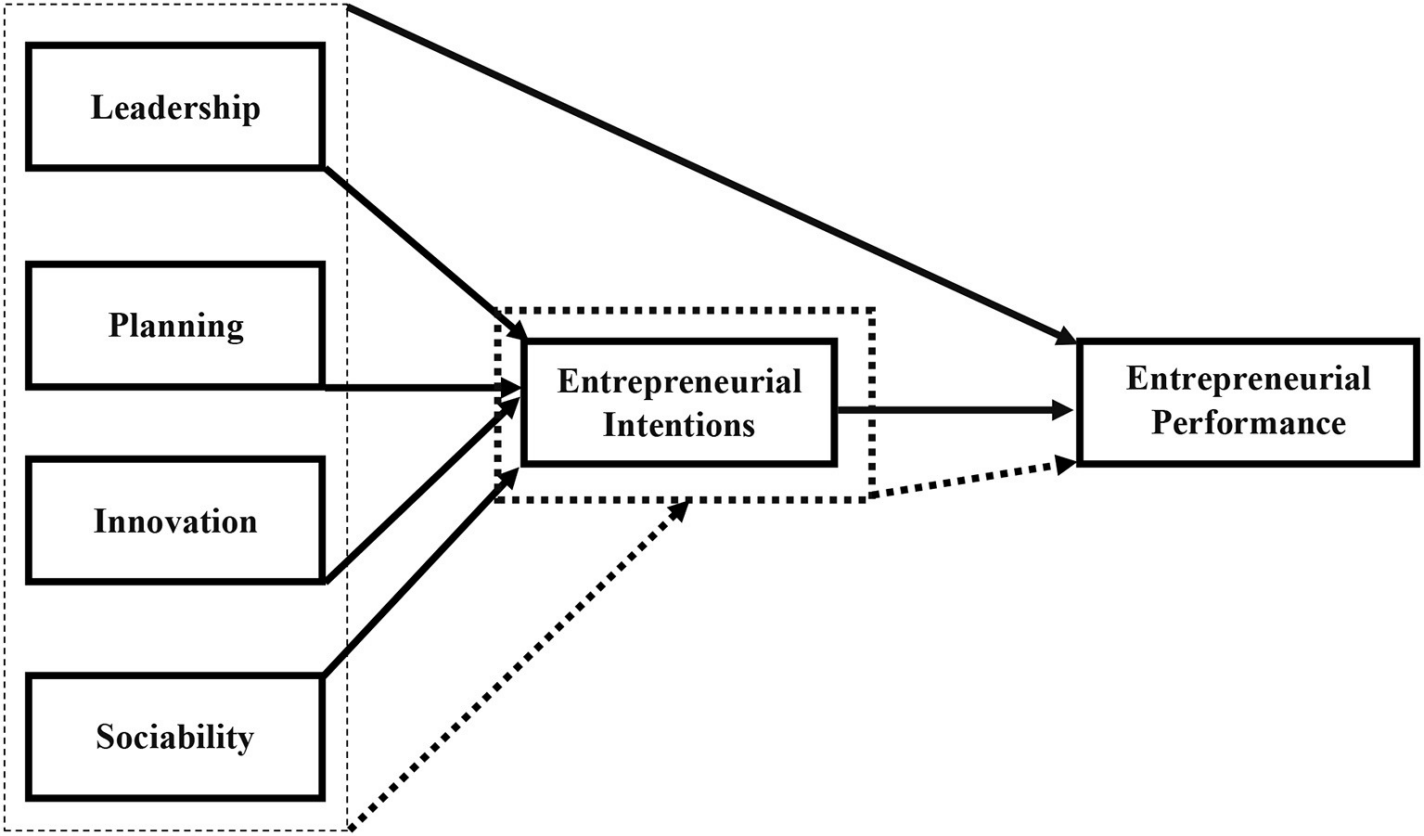
Conceptual model.

The earlier model is the representation of the proposed conceptual framework based on earlier literature gaps. Therefore, this model proposes, as given in Figure 1, a total of 13 hypotheses which are as follows:

*H1*: *Leadership has a positive relationship with entrepreneurial intentions*.
*H2*: *Planning has a positive relationship with entrepreneurial intentions*.
*H3*: *Innovation has a positive relationship with entrepreneurial intentions*.
*H4*: *Sociability has a positive relationship with entrepreneurial intentions*.
*H5*: *Leadership has a positive relationship with entrepreneurial performance*.
*H6*: *Planning has a positive relationship with entrepreneurial performance*.
*H7*: *Innovation has a positive relationship with entrepreneurial performance*.
*H8*: *Sociability has a positive relationship with entrepreneurial performance*.
*H9*: *Entrepreneurial intentions have a positive relationship with entrepreneurial performance*.
*H10*: *Entrepreneurial intentions mediate the relationship between leadership and entrepreneurial performance of vloggers*.
*H11*: *Entrepreneurial intentions mediate the relationship between planning and entrepreneurial performance of vloggers*.
*H12*: *Entrepreneurial intentions mediate the relationship between innovation and entrepreneurial performance of vloggers*.
*H13*: *Entrepreneurial intentions mediate the relationship between sociability and entrepreneurial performance of vloggers*.

## Research Methodology

### Measurement Scale for Constructs

This study considered variables used in the earlier well-known study. The measurements or items were also considered from an earlier study, which were used in data collection via questionnaires. Among the total number of items, four items were considered for entrepreneurial characteristics (i.e., innovation, leadership, planning, and sociability; Moraes et al., 2018), five items for entrepreneurial performance (Hallak et al., 2014), and four items for entrepreneurial intentions (Thompson, 2009). The overall 5-point Likert scale was considered in this study from 1 strongly disagree to 5 strongly agree.

### Procedure of Sampling and Data Collection

This study is cross-sectional in nature, the data were collected through primary resources by using an adapted questionnaire, and 128 entrepreneurial vloggers of India were considered. The criteria used were the following: being a respondent, to which vlogging category they belong, spending more time vlogging, and like to take initiative for better performance. This criteria questionnaire was designed, and 150 responses were collected by using an online survey, but responses of 128 participants were finalized for the analysis.

## Data Analysis And Results

**Figure 2:**
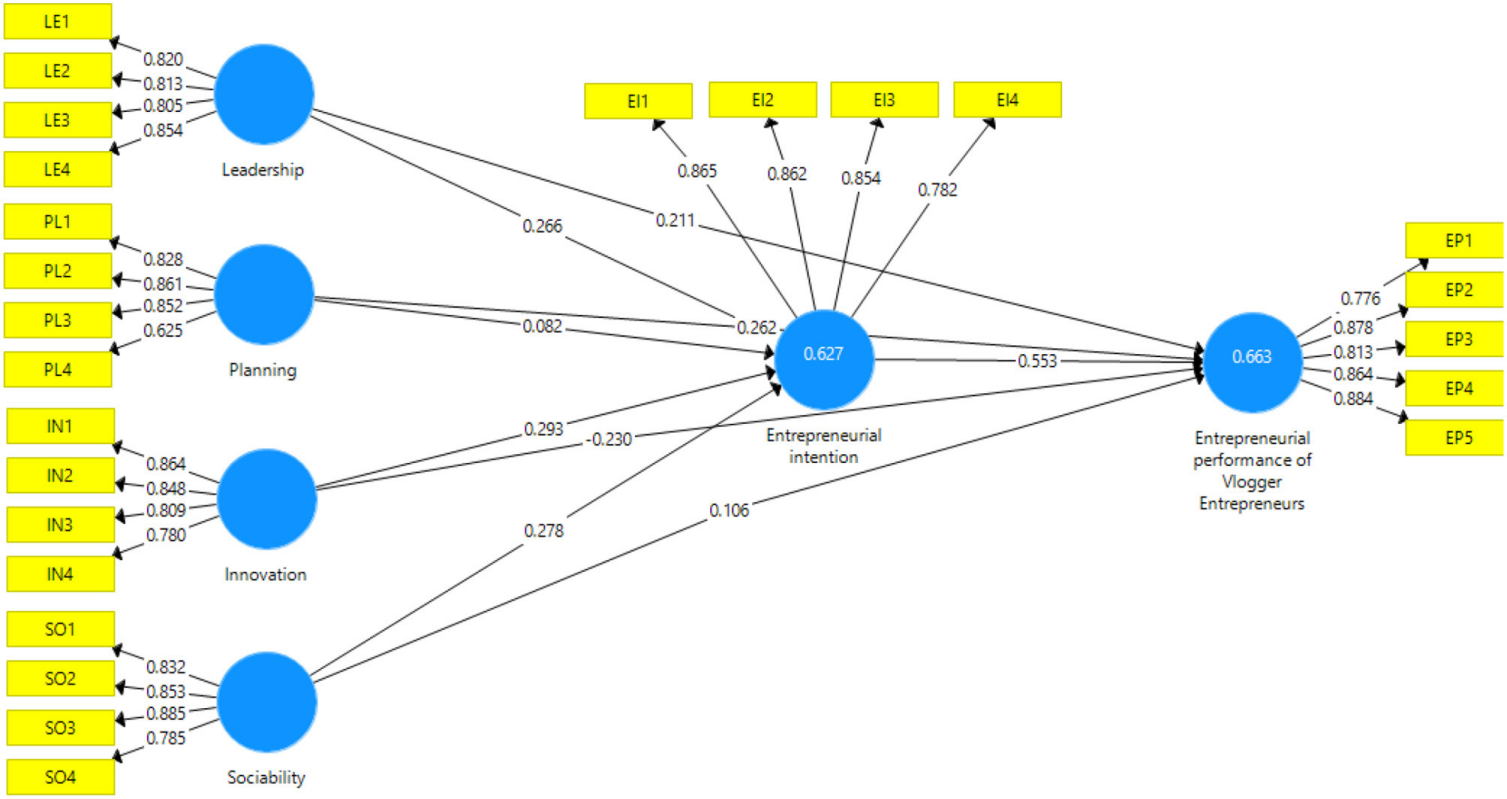
SmartPLS algorithm outcomes.

**Figure 3:**
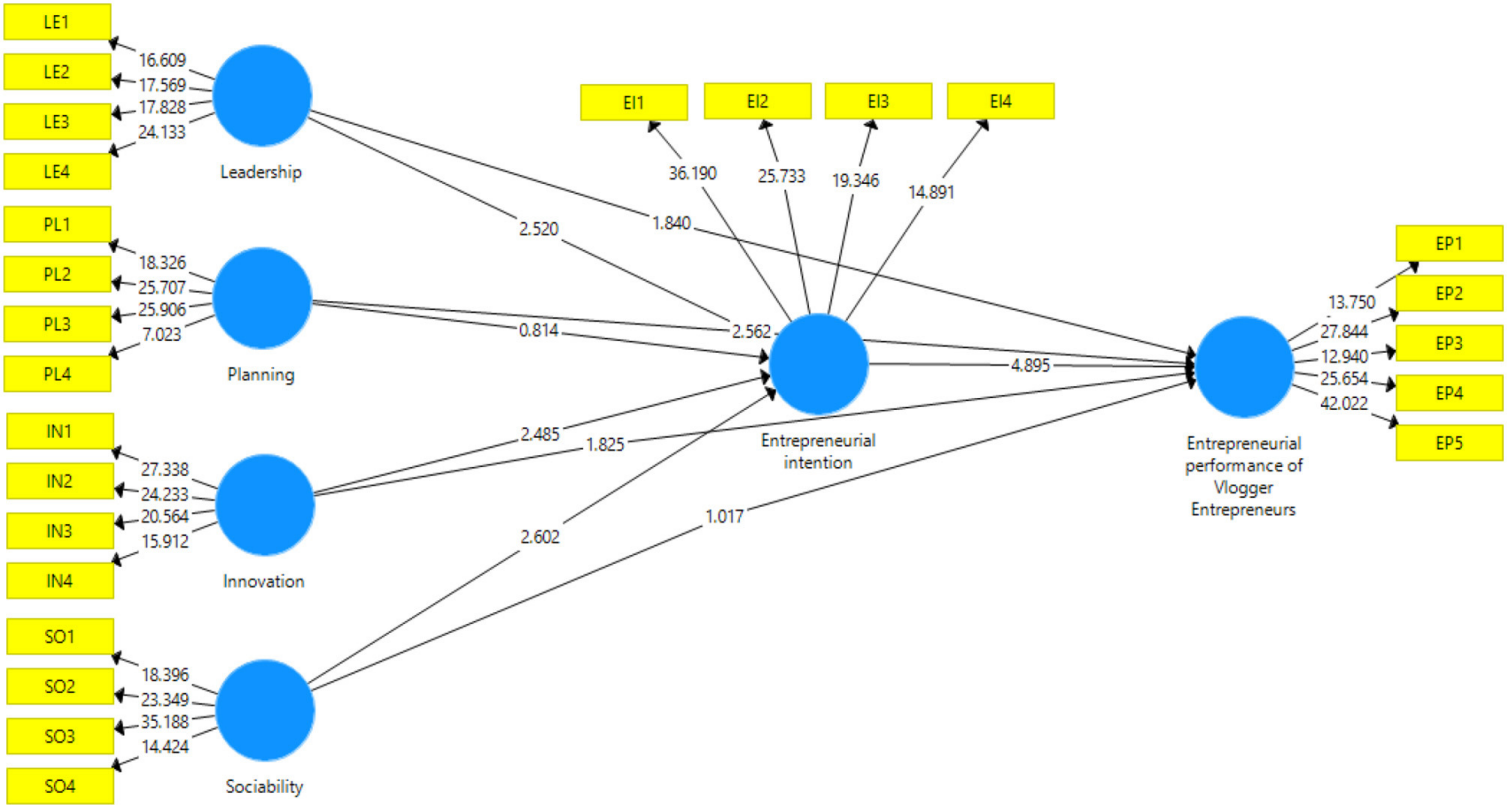
SmartPLS bootstrapping outcomes.

This study used the partial least square structural equation modeling (PLS-SEM) to examine the proposed conceptual model via using the SmartPLS version 3.3.3 application. The approach consists of two parts, namely, (a) assessment of measurement model and (b) assessment of structural model. These two steps are often traded off using a single-step approach as advised by the earlier studies. The assessment of the measurement model explains the measurements of all variables in the model, and the assessment of the structural model identifies the relationship among variables in the model, details given in Figures 2, 3.

The demographic summary is illustrated in Table 1. The process of data collection had five demographic questions. The overall summary demonstrated that males and females are similarly involved in vlogging and starting their journey as vlogger entrepreneurs. Also, the demographic summary highlighted the top eight currently trending vlogging categories in which Indian entrepreneurial vloggers are interested. Around 90% of surveyed vloggers are interested to do what they are doing as entrepreneurial vloggers, and around 62% see a bright future as an entrepreneur in vlogging as a business. The majority of surveyed entrepreneurial vloggers believed that any unique idea that is the core of entrepreneurship can help to boost the performance and can attract the audience than any already prevailed idea.

**Table 1:** Demographic details.

	**Frequency**	**%**
**Gender**		
Yes	62	48.44
No	66	51.56
**What is your category of vlogging?**		
Makeup	10	7.81
Gaming	13	10.16
Food	11	8.59
Fitness	16	12.50
Unboxing	16	12.50
Tech reviews	18	14.06
Travel	19	14.84
Comedy	25	19.53
**Do you love doing what you do in your vlogs?**		
Yes	114	89.06
No	14	10.94
**How many hours do you work to do and manage to vlog?**		
1 h or less	21	16.41
2–3 h	23	17.97
4–5 h	25	19.53
6–7 h	31	24.22
8 h or above	28	21.88
**Do you see a bright future in vlogging?**		
Yes	80	62.50
No	48	37.50
**Do you think, a unique idea of vlogging can enhance vlogging performance and attract the audience?**		
Yes	109	85.16
No	19	14.84

*n = 128*.

### Descriptive Statistics and Assessment of Measurement Model

The descriptive statistic gives an overview of the distribution type of the data. The mean ranges from 3.8495 to 4.1328 as this study considered the 5-point Likert scale. Also, the standard deviation ranges from 0.901 to 1.061.

The assessment of the measurement model is mainly based on analyzing the reliability, convergent, and discriminant validity of constructs and measurements. This study used the Cronbach's alpha to determine the reliability of all constructs in this study. Hence, the value of Cronbach's alpha is between 0.803 and 0.899; therefore, it exceeds the threshold point of 0.700 (Hair et al., 2017). Moreover, each CR coefficient falls between 0.873 and 0.925, and thus exceeds the threshold point of 0.700 (Hair et al., 2017). Thus, the requirement of construct reliability (CR) has been met and satisfied, and the Cronbach's alpha and CR coefficients are well above the threshold point. It can be stated that all the constructs are error-free in terms of the reliability of the constructs in the model. Similarly, the reliability of indicators or the items within the constructs is measured through the factor loadings (Isaac et al., 2019). The coefficients of up to 0.700 or above demonstrate meaningful factor loadings (Hair et al., 2017). Therefore, Table 2 illustrates that every factor loading is above the threshold point, although PL4 is 0.624 which is above 0.600; therefore, it can also fulfill the requirement (Hair et al., 2010; Ul-Haq et al., 2020).

**Table 2:** Measurement model and descriptive statistics.

**Constructs**	**Code**	**FD**	**Cronbach's α**	**CR**	**AVE**	**M**	***SD***
Innovation			0.844	0.895	0.682	3.8495	1.011
	IN1	0.864					
	IN2	0.848					
	IN3	0.809					
	IN4	0.780					
Leadership			0.842	0.894	0.678	4.0095	0.971
	LE1	0.820					
	LE2	0.813					
	LE3	0.805					
	LE4	0.854					
Planning			0.803	0.873	0.636	4.02925	1.059
	PL1	0.828					
	PL2	0.861					
	PL3	0.852					
	PL4	0.625					
Sociability			0.86	0.905	0.705	3.78325	1.061
	SO1	0.832					
	SO2	0.853					
	SO3	0.885					
	SO4	0.785					
Entrepreneurial intention			0.862	0.906	0.708	4.03525	0.901
	EI1	0.865					
	EI2	0.862					
	EI3	0.854					
	EI4	0.782					
Entrepreneurial performance of vlogger entrepreneurs			0.899	0.925	0.712	4.1328	0.909
	EP1	0.776					
	EP2	0.878					
	EP3	0.813					
	EP4	0.864					
	EP5	0.884					

*FD, factor loadings; CR, construct reliability; AVE, average variance extracted; and α, Cronbach's alpha*.

Furthermore, the AVE is considered to estimate the convergent validity of the constructs; it measures the positive association between the items of the constructs of identical constructs (Isaac et al., 2019).

Previously, the value of each AVE falls between 0.656 and 0.712; therefore, it is well above the threshold point of 0.500 (Sarstedt et al., 2017; Haq and Awan, 2020). Thus, the convergent validity is satisfactory for all variables in the model.

Finally, the Fornell and Larcker criterion and heterotrait–monotrait ratio of correlation (HTMT) are considered to test the discriminant validities. In Table 3, the coefficients demonstrated the satisfactory discriminant validity. The diagonal values in Table 3 are greater than the below mentioned values, as conditionally, the higher diagonal values produced by Fornell and Larcker ratio than their below mentioned values demonstrate a strong correlation among constructs. Similarly, the HTMT values are also under the ratio as mentioned by Lia et al. (2020), which is 0.85 as the values around 0.90 is the indication of error. Hence, all the values in Table 4 are within the range and below 0.85, and thus HTMT outcomes also provide satisfactory evidence for discriminant validity.

**Table 3:** Fornell–Larcker ratio criterion.

	**EI**	**EP**	**IN**	**LE**	**PL**	**SO**
EI	0.842					
EP	0.749	0.844				
IN	0.72	0.528	0.826			
LE	0.667	0.668	0.649	0.823		
PL	0.565	0.646	0.546	0.678	0.797	
SO	0.690	0.566	0.753	0.558	0.512	0.839

*EI, entrepreneurial intentions; EP, entrepreneurial performance; IN, innovation; LE, leadership; PL, planning; SO, sociability*.

**Table 4:** HTMT ratio of correlation.

	**EI**	**EP**	**IN**	**LE**	**PL**	**SO**
EI						
EP	0.847					
IN	0.845	0.599				
LE	0.780	0.763	0.768			
PL	0.684	0.748	0.680	0.824		
SO	0.798	0.636	0.883	0.651	0.624	–

*EI, entrepreneurial intentions; EP, entrepreneurial performance; IN, innovation; LE, leadership; PL, planning; SO, sociability*.

### Assessment of Structural Model

The assessment of the structural model involves the estimation of beta (β), respective *p*-values, and *t*-statistics through the bootstrap technique with 5,000 resamples in the SmartPLS 3.3.3 application. The *p*-values and *t*-statistics are used to analyze the statistical significance, and *Q*^2^ demonstrates the predictive accuracy. Tables 5, 6 illustrated the outcomes of the structural model in the form of the path analysis with direct and indirect effects, respectively. The first entrepreneurial characteristic “leadership” has meaningful impact on the entrepreneurial intentions that are confirmed with *t* − *value* = 2.525:*p*−*value* = 0.006. The second entrepreneurial characteristic “planning” also has no meaningful impact on entrepreneurial intentions under *t* − *value* = 0.821:*p*−*value* = 0.206, and thus H2 is rejected. The third entrepreneurial characteristic “innovation” meaningfully predicts the entrepreneurial intentions with *t* − *value* = 2.509:*p*−*value* = 0.006, and thus H3 is supported. Likewise, the fourth entrepreneurial characteristic “sociability” also has a positive meaningful impact on entrepreneurial intentions with *t* − *value* = 2.562:*p*−*value* = 0.005. In a similar fashion, three out of four entrepreneurial characteristics, namely, leadership, planning, and innovation, have a positive meaningful impact on entrepreneurial performance of an entrepreneurial vlogger, which is statistically proved under *t* − *value* = 1.815:*p*−*value* = 0.035, *t* − *value* = 2.508:*p*−*value* = 0.006, and *t* − *value* = 1.826:*p*−*value* = 0.034, respectively.

**Table 5:** Direct effects.

**Hypothesis**	**Paths**	**(O)**	**(M)**	**(STDEV)**	**T statistics**	***P*-values**	**Q2**
H1	LE -> EI	0.266	0.265	0.105	2.525	0.006	0.419
H2	PL -> EI	0.082	0.079	0.099	0.821	0.206	
H3	IN -> EI	0.293	0.305	0.117	2.509	0.006	
H4	SO -> EI	0.278	0.270	0.109	2.562	0.005	
H5	LE -> EP	0.211	0.212	0.117	1.815	0.035	0.441
H6	PL -> EP	0.262	0.255	0.104	2.508	0.006	
H7	IN -> EP	−0.230	−0.210	0.126	1.826	0.034	
H8	SO -> EP	0.106	0.102	0.105	1.009	0.156	
H9	EI -> EP	0.553	0.539	0.114	4.850	0.000	

*O, original sample; M, sample mean; STDEV, standard deviation; EI, entrepreneurial intentions; EP, entrepreneurial performance; IN, innovation; LE, leadership; PL, planning; SO, sociability*.

**Table 6:** Indirect effects.

**Hypothesis**	**Paths**	**(O)**	**(M)**	**(STDEV)**	**T statistics**	***P*-values**
H10	LE -> EI -> EP	0.147	0.145	0.068	2.155	0.016
H11	PL -> EI -> EP	0.045	0.044	0.055	0.824	0.205
H12	IN -> EI -> EP	0.162	0.161	0.065	2.496	0.006
H13	SO -> EI -> EP	0.154	0.147	0.070	2.185	0.014

*O, original sample, M, sample mean, STDEV, standard deviation, EI, entrepreneurial intentions, EP, entrepreneurial performance, IN, innovation, LE, leadership, PL, planning, SO, sociability*.

In contrast, the sociability relationship with entrepreneurial performance of an entrepreneurial vlogger was proved insignificant, where *t* − *value* = 1.009:*p*−*value* = 0.156. The last direct effect is also confirmed as the entrepreneurial intentions have a meaningful positive impact on the entrepreneurial performance of entrepreneurial vloggers with *t* − *value* = 4.850:*p*−*value* = 0.000.

Talking about the indirect effects illustrated in Table 6, the indirect effects confirmed the proposed mediation effects. Four mediations were proposed, and out of these four, three of them were confirmed and supported. The mediating effect of entrepreneurial intentions was proved significant between three entrepreneurial characteristics, namely, “leadership, innovation, and sociability,” and entrepreneurial performance of entrepreneurial vloggers, and thus H10, H12, and H13 are confirmed. The coefficient of all three indirect variables given as *t* − *value* = 2.151:*p*−*value* = 0.016, *t* − *value* = 2.496:*p*−*value* = 0.006, and *t* − *value* = 2.185:*p*−*value* = 0.014, respectively. On the other hand, the mediating role of entrepreneurial intentions proved insignificant where *t* − *value* = 0.824:*p*−*value* = 0.205, and thus H11 is rejected.

## Discussion Of Findings

This study explores the association of the individual personality entrepreneurial characteristics of an entrepreneur among Indian vloggers. Four entrepreneurial characteristics are considered as independent variables, the performance of entrepreneurial vloggers is considered as the dependent variable, and entrepreneurial intentions are considered as a mediator in current settings. The research established that entrepreneurial characteristics deliver a positive significant impact on the entrepreneurial intentions and the overall performance of the entrepreneurial vloggers, which implies that the characteristics of individual entrepreneurs have a positive impact on the overall performance of entrepreneurial vloggers and the entrepreneurial intentions. Additionally, it delineates that entrepreneurial vloggers, who have entrepreneurial characteristics like leadership, innovation, and sociability, can improve their overall performance. The more innovative entrepreneurial vlogger will lead to an increase in the overall performance. Likewise, vloggers who have leadership characteristics will have more improved and better performance as entrepreneurial vloggers. Not only leadership and innovative personal traits can make a difference, but planning has also led entrepreneurial vloggers as better-performing individuals. The sociability showed an insignificant association, which may be due to the reason that people do not pay much attention toward entrepreneurial vloggers at the beginning or even make fun of them with friends until they get succeed and have a well-reputed channel or network. That is one of the reasons that sociability stood insignificant.

All entrepreneurial characteristics positively predict the entrepreneurial intentions of vloggers. Specifically, the demonstration of a positive association of leadership, planning, and innovations with entrepreneurial intentions implies that if any vlogger has these characteristics, then these may be useful to predict and enhance the entrepreneurial intentions in the entrepreneurial vloggers of India. In contrast, the insignificant association among planning as entrepreneurial characteristic and overall intentions as an entrepreneurial vlogger established that planning may not be a suitable characteristic in entrepreneurial vloggers in Indian settings. There may be because many vlogging videos are unplanned and do not pay much attention to planning and memorizing the script. Moreover the lengthy video of vlogging cannot be memorized and shared with people. Therefore, planning traits may lack and have an insignificant relationship with the intentions of entrepreneurial vloggers. These findings imply that entrepreneurial characteristics can enhance the overall performance of the entrepreneurial vloggers and also can be a strong source for enhancing entrepreneurial intentions. Therefore, vloggers are needed to pay strict attention to their personalities as personality traits can increase or decrease the performance in vlogging in Indian settings.

## Conclusion

The current technological revolution via the Internet has a crucial impact on business opportunities for entrepreneurs. Likewise, Internet availability has led to many business opportunities, and vlogging is one among them. Vloggers are initiating their channels and becoming entrepreneurs. Interestingly, the personality traits of vloggers play a vital role in their success and overall performance. This study explores the entrepreneurial characteristics of vloggers and entrepreneurial intentions and performance of entrepreneurial vloggers in India. This study considered entrepreneurial characteristics and studied the impact of each characteristic individually on the performance of entrepreneurial vloggers. Additionally, entrepreneurial intentions are considered as a mediator in the study. The overall research philosophy is positivism, and a deductive approach is employed. The data were collected through online social media platforms from the entrepreneur vloggers. For the purpose of the analysis, this study considered SmartPLS to estimate the SEM two-step analysis approach for estimation. The overall findings revealed that the proposed research model is effective to meaningfully predict and measure the relationship between entrepreneurial characteristics and entrepreneurial intentions and the performance of entrepreneurial vloggers. Moreover, the mediating role of entrepreneurial intentions is proved partially mediated between the relationship of entrepreneurial characteristics and the performance of entrepreneurial vloggers. The direct effect of planning on entrepreneurial intentions has no predictive ability and is statistically insignificant; likewise, the mediating role of entrepreneurial intentions is also insignificant in this relationship. Also, the sociability characteristic could not predict the performance of entrepreneurial vloggers. In overview, these findings are very much crucial for the entrepreneurial vloggers to analyze their characteristics toward the overall performance. It may help vloggers to add characteristics to enhance the overall vlogging performance.

### Limitations and Research Direction

This study also has several limitations: it is cross-sectional and firmly based on the responses of the respondents. Moreover, this study is conducted in the Indian settings, where India is a developing country; therefore, these findings cannot be generalized for the settings of other countries, particularly for developed countries such as the United States and the United Kingdom. Therefore, more studies are required to generalize these findings in vlogging strand. Several implications can be supportive toward vlogging trend, and limitations of these studies can be a roadmap for future scholars to explore the area. The authors recommend the role of the VIRO framework as mediators as suggested by Ul-Haq et al. (2020) for future research.

## Data Availability Statement

The original contributions presented in the study are included in the article/supplementary material, further inquiries can be directed to the corresponding author/s.

## Author Contributions

QX and LL: initial and final draft. HM, SC, and PC: analysis, data collection, and review. All authors contributed to the article and approved the submitted version.

## Conflict of Interest

The authors declare that the research was conducted in the absence of any commercial or financial relationships that could be construed as a potential conflict of interest.

## Publisher's Note

All claims expressed in this article are solely those of the authors and do not necessarily represent those of their affiliated organizations, or those of the publisher, the editors and the reviewers. Any product that may be evaluated in this article, or claim that may be made by its manufacturer, is not guaranteed or endorsed by the publisher.
